# Precision determination of the strong coupling constant within a global PDF analysis: NNPDF Collaboration

**DOI:** 10.1140/epjc/s10052-018-5897-7

**Published:** 2018-05-24

**Authors:** Richard D. Ball, Stefano Carrazza, Luigi Del Debbio, Stefano Forte, Zahari Kassabov, Juan Rojo, Emma Slade, Maria Ubiali

**Affiliations:** 1 The Higgs Centre for Theoretical Physics, University of Edinburgh, JCMB, KB, Mayfield Rd, Edinburgh, Scotland EH9 3JZ UK; 2 Theoretical Physics Department, CERN, 1211 Geneva, Switzerland; 3 Tif Lab, Dipartimento di Fisica, Università di Milano and INFN, Sezione di Milano, Via Celoria 16, 20133 Milan, Italy; 4 Cavendish Laboratory, University of Cambridge, Cambridge, CB3 0HE UK; 5 Department of Physics and Astronomy, VU University, 1081 HV Amsterdam, The Netherlands; 6 Nikhef Theory Group, Science Park 105, 1098 XG Amsterdam, The Netherlands; 7 Rudolf Peierls Centre for Theoretical Physics, University of Oxford, 1 Keble Road, Oxford, OX1 3NP UK; 8 DAMTP, University of Cambridge, Wilberforce Road, Cambridge, CB3 0WA UK

## Abstract

We present a determination of the strong coupling constant $\alpha _s(m_Z)$ based on the NNPDF3.1 determination of parton distributions, which for the first time includes constraints from jet production, top-quark pair differential distributions, and the *Z* $p_T$ distributions using exact NNLO theory. Our result is based on a novel extension of the NNPDF methodology – the correlated replica method – which allows for a simultaneous determination of $\alpha _s$ and the PDFs with all correlations between them fully taken into account. We study in detail all relevant sources of experimental, methodological and theoretical uncertainty. At NNLO we find $\alpha _s(m_Z) = 0.1185 \pm 0.0005^\text {(exp)}\pm 0.0001^\text {(meth)}$, showing that methodological uncertainties are negligible. We conservatively estimate the theoretical uncertainty due to missing higher order QCD corrections (N$^3$LO and beyond) from half the shift between the NLO and NNLO $\alpha _s$ values, finding $\Delta \alpha ^\mathrm{th}_s =0.0011$.

## Introduction

The value of the strong coupling constant $\alpha _s\left( m_Z \right)$ is a dominant source of uncertainty in the computation of several LHC processes. This uncertainty is often combined with that on parton distributions (PDFs), with which it is strongly correlated. However, while PDF uncertainties have reduced considerably over the years, as it is clear for example by comparing the 2012 [1] and 2015 [2] PDF4LHC recommendations, the uncertainty on the $\alpha _s$ PDG average [3] remains substantially unchanged since 2010 [4]. As a consequence, the uncertainty on $\alpha _s$ is now the dominant source of uncertainty for several Higgs boson production cross-sections [5].

Possibly the cleanest [6, 7] determinations of $\alpha _s$ come from processes that do not require a knowledge of the PDFs, such as the global electroweak fit [8]. These are free from the need to control all sources of bias which may affect the PDF determination and contaminate the resulting $\alpha _s$ value. A determination of $\alpha _s$ jointly with the PDFs, however, has the advantage that it is driven by the combination of a large number of experimental measurements from several different processes. This is advantageous because possible sources of uncertainties related to specific measurements, either of theoretical or experimental origin, are mostly uncorrelated amongst each other and will average out to some extent in the final $\alpha _s$ result. In addition to the above, the simultaneous global fit of $\alpha _s$ and the PDFs is likely to be more precise and possibly also more accurate than individual determinations based on pre-existing PDF sets, many of which have recently appeared [9–15]. This is due to the fact that it fully exploits the information contained in the global dataset while accounting for the correlation of $\alpha _s$ with the underlying PDFs.

**Fig. 1:**
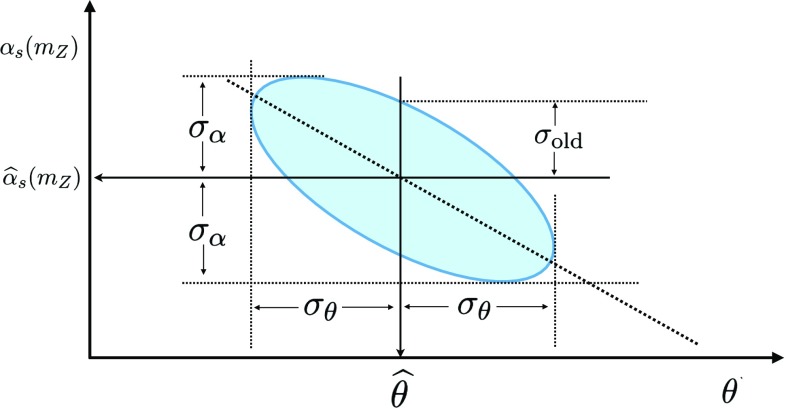
Comparison between the standard deviation of a pair of correlated variables $(\alpha _s,\theta )$ and the one-sigma range for the variable $\alpha _s$ along the best-fit line of $\theta$. The best fit is denoted as $(\hat{\alpha }_s,\hat{\theta })$ and the ellipse is the one-sigma contour about it. The standard deviations on $(\alpha _s,\theta )$ are $(\sigma _{\alpha },\sigma _\theta )$, while $\sigma _\mathrm{old}$ is the one-sigma interval for $\alpha _s$ with fixed $\theta =\hat{\theta }$

Here we present a determination of $\alpha _s$ which exploits the most recent PDFs obtained with the NNPDF methodology, namely NNPDF3.1 [16]. This updates a previous determination of $\alpha _s$ [17, 18] based on NNPDF2.1 [19, 20]. In comparison to this previous PDF set, NNPDF3.1 represents a substantial improvement both in terms of input dataset, theoretical calculations, and fitting methodology. Specifically, NNPDF3.1 is the first PDF set to make such an extensive use of LHC data as to be dominated by them. It is in fact the first global analysis to simultaneously use differential top, inclusive jet, and *Z* $p_T$ distribution data, all using exact NNLO theory. Indeed, typical PDF uncertainties are of order of one to three percent in the data region for NNPDF3.1, about a factor two smaller than they were for NNPDF2.1.

This greater precision in the PDF determination requires a corresponding improvement in the methodology used for the $\alpha _s$ extraction. In our previous work [17, 18], PDF replicas were determined for a number of fixed values of $\alpha _s$, which was then extracted from the $\chi ^2$ profile versus $\alpha _s$ of the best fit PDF, obtained as an average over the replicas. Here instead, both $\alpha _s$ and PDFs are determined from a simultaneous minimization in their combined parameter space. As we will discuss below, this new method corresponds roughly to determining the value and uncertainty on $\alpha _s$ from the error ellipse of the multivariate measurement in the $\left( \alpha _s, \mathrm{PDF}\right)$ hyperspace, and the old method corresponds to performing a scan of $\alpha _s$ along the best-fit PDF line, see Fig. 1 for a schematic illustration. In a situation when the variables are highly correlated, especially if the semi-axes of the ellipse are of very different length, the procedure used in our previous work might lead to an underestimate of the uncertainty in $\alpha _s$. Hence the new procedure becomes very relevant, now that some PDF uncertainties are rather small.

It turns out that the implementation of this simultaneous minimization within the NNPDF methodology is nontrivial: it requires the development of a suitable generalization of the standard NNPDF approach, which we call the correlated replica method. Using this strategy, $\alpha _s$ can be treated like any other quantity that depends on the PDFs. In particular, its central value and uncertainty can be determined by performing statistics over a replica sample. This means that, for example, the uncertainty on $\alpha _s$ is the standard deviation of an ensemble of $\alpha _s$ values. As we shall see, this allows for a determination of $\alpha _s$ with small experimental uncertainties, and negligible methodological uncertainties. Having reduced very much the size of all other uncertainties, the problem of accurately estimating theoretical uncertainties becomes quite serious. This is specifically problematic in the case of missing higher-order uncertainties (MHOUs), for which no fully satisfactory method has been developed. Here we will conservatively estimate the theoretical uncertainty due to missing higher order QCD corrections (N$^3$LO and beyond) from half the shift between the NLO and NNLO $\alpha _s$ values.

This paper consists of two main parts. First, in Sect. 2 we present the correlated replica method used for the determination of $\alpha _s$, explain how it is used to estimate the associated PDF uncertainties, and compare it with the method used in previous NNPDF determinations. Then, in Sect. 3 we present our determination of $\alpha _s$ at NLO and NNLO together with a careful assessment of all sources of uncertainty. Possible future developments are briefly outlined in Sect. 4.

## The correlated Monte Carlo replica method

As discussed in the introduction, the $\alpha _s$ determination presented here differs from our previous one [17, 18] because now the value of $\alpha _s$ and its uncertainty are determined from a correlated fit together with the PDFs. After briefly summarizing the main aspects of the NNPDF methodology and the way it was used to determine $\alpha _s$ in Ref. [17, 18], we describe the main idea of the new method, and then discuss the details of its implementation. Only the salient aspects of the NNPDF methodology will be recalled here; the reader is referred to the original literature (see Ref. [16], of which we follow the notation, and references therein) and recent reviews [2, 21, 22] for a more detailed discussion.

### General strategy

The NNPDF fitting methodology is based on constructing a Monte Carlo representation of the original data sample consisting of pseudodata (Monte Carlo replicas of the original data), and fitting PDF replicas to these data replicas. Specifically, starting with an $$N_\mathrm{dat}$$-component vector of experimental points *D* with components $$D_i$$, a set of $$N_\mathrm {rep}$$ replicas $$ D^{(k)}$$ of the data is generated by means of:

2.1 $$\begin{aligned} D_i^{(k)}= & {} \left( 1 + r_i^{\mathrm{nor},k} \sigma _i^\mathrm{nor}\right) \nonumber \\&\times \left( D_i + \sum _{p=1}^{N_\mathrm{sys}} r_{i,p}^{\mathrm{sys},k} \sigma ^\mathrm{sys}_{i,p} + r_i^{\mathrm{stat},k} \sigma ^\mathrm{stat}_i\right) ,\nonumber \\&\quad i=1,\ldots ,N_\mathrm{dat}, \end{aligned}$$

where $k=1,\ldots ,N_\mathrm{rep}$; $\sigma _i^\mathrm{nor}$, $\sigma _i^\mathrm{sys}$ and $\sigma _i^\mathrm{stat}$ are normalization, systematic and statistical uncertainties, and $r_i$ are random numbers such that statistics over the replica sample reproduces the original statistical properties of the data in the limit of large $N_\mathrm {rep}$. For example, this means that

2.2 $$\begin{aligned} \lim _{N_\mathrm{rep}\rightarrow \infty } \mathrm {cov}\left( D_i D_j\right) =C_{ij}, \end{aligned}$$

where $\mathrm {cov}$ denotes the covariance over the replica sample and $C_{ij}$ is the full experimental covariance matrix of the data.

A PDF replica is then fitted to each data replica $$ D^{(k)}$$. In the NNPDF approach, PDFs are parametrized using neural networks, in turn specified by a vector of parameters $$\theta $$. In the most recent NNPDF3.1 analysis, this vector $$\theta $$ has 296 components, corresponding to 37 parameters for eight neural networks (for the up, antiup, down, antidown, strange, antistrange, total charm and gluon PDFs). Thus, for each data replica $$ D^{(k)}$$ a best-fit $$\theta ^{(k)}$$ is found by minimizing a figure of merit characterizing the agreement between theory and data:

2.3 $$\begin{aligned} \chi ^2(\theta ,D) = \frac{1}{N_\mathrm{dat}} \sum _{i,j} (T_{i}[\theta ] - D_{i}) \, \left( C_{t_0}^{-1}\right) _{ij} \, (T_{j}[\theta ] - D_{j} ). \end{aligned}$$

Here, $T_{i}[\theta ]$ is the theoretical prediction for the *i*th datapoint, dependent on the set of parameters $\theta$, and $C_{t_0}$ is the covariance matrix used in the fit. Recall that in the presence of multiplicative uncertainties, $C_{t_0}$ cannot be directly identified with the experimental covariance matrix *C* used for pseudodata generation Eq. (2.1) lest the fit be biased [23], and must thus be constructed instead using a suitable procedure such as the $t_0$ method [24] (see also [25]).

A peculiarity of the NNPDF approach is that the best-fit parameters of each replica, $\theta ^{(k)}$, are not defined as the absolute minimum of the $\chi ^2$ Eq. (2.3) in order to avoid overfitting, i.e. in order not to fit statistical fluctuations. Instead, a suitable cross-validation algorithm is employed [26]. We thus obtain a set of best-fit PDF replicas $\theta ^{(k)}$, each determined as the minimum with respect to $\theta$ of the figure of merit ${\chi ^{2(k)}}$ computed using the *k*th data replica:

2.4 $$\begin{aligned} \theta ^{(k)}&=\mathrm {argmin}\left[ \chi ^2(\theta ,D^{(k)})\right] , \end{aligned}$$

where $$\mathrm {argmin}$$ should be understood as minimization through cross-validation, rather than as the absolute minimum. Note that, because we employ non-deterministic minimization algorithms, specifically genetic algorithms, the best-fit $$\theta ^{(k)}$$ corresponding to a given data replica $$ D^{(k)}$$ is not unique; two identical data replicas $$ D^{(k_1)}=D^{(k_2)}$$ may lead to two different $$\theta ^{(k_1)}\not =\theta ^{(k_2)}$$ in two runs of the minimization algorithm.

In summary, the standard NNPDF methodology produces a set of replicas $$ D^{(k)}$$ of the original data, and uses them to construct a set of PDF replicas which correspond to parameters $$\theta ^{(k)}$$, where *k* runs over the replica sample.

The theory predictions $T_i$, which enter in the figure of merit of the fit Eq. (2.3) depend not only on the PDF parameters $\theta$, but also on theory parameters, specifically the value of $\alpha _s$. Therefore, in general we can view the figure of merit as a function $\chi ^2(\alpha _s,\theta ,D)$. In standard NNPDF determinations, $\alpha _s$ is treated as a fixed parameter, along with all other theory parameters, such as quark masses, CKM matrix elements, the fine structure constant, and so on. On the other hand, it is well known (see e.g. Ref. [27] for an early reference) that PDFs are strongly correlated to the value of $\alpha _s$, so a determination of the combined PDF+$\alpha _s$ uncertainty on a process which depends on both, requires knowledge of the PDFs as $\alpha _s$ is varied. With this motivation, NNPDF sets are routinely released for different fixed values of $\alpha _s$, where the procedure of generating data replicas $D^{(k)}$ and determining PDF replicas determined by the best-fit parameters $\theta ^{(k)}$ is repeated several times for different values of $\alpha _s$.

In our previous work [17, 18], $\alpha _s$ was determined by first producing PDF fits for a range of values of $\alpha _s$. The $\chi ^2(\alpha _s)$ of the mean of all the replicas was then fitted to a parabola as a function of $\alpha _s$. This methodology has two main drawbacks. The first is that, as mentioned, the PDFs are strongly correlated to the value of $\alpha _s$. With this method, however, the $\chi ^2$ profile is determined as a function of $\alpha _s$ along the line in $\theta$ space which corresponds to the best-fit $\theta$ at each particular value of $\alpha _s$, without taking into account the variations in $\theta$ space. Hence, as illustrated in Fig. 1, with the methodology of Refs. [17, 18] the resulting uncertainty on $\alpha _s$ could be somewhat underestimated.

The second drawback is more subtle. In the NNPDF procedure, the PDF uncertainty is determined from statistics over the replica sample, so a one-sigma interval is determined by computing a standard deviation over replicas. Whether or not this corresponds exactly to a one-sigma (i.e. $\Delta \chi ^2 =1$) interval in $\alpha _s$ space is unclear. In fact, in PDF determinations based on Hessian minimization in parameter space, the $\Delta \chi ^2 =1$ criterion is modified by a suitable tolerance factor [28–30], which possibly accounts for data inconsistencies or parametrization bias. It is unclear, but certainly possible, that PDF uncertainties estimated in the NNPDF fits also include, at least to some extent, such a tolerance.

Ideally, we would like a method of determining $\alpha _s$ in which the uncertainty on $\alpha _s$ is determined on exactly the same footing as the PDF uncertainty, and which thus yields the full probability distribution for $\alpha _s$, marginalised with respect to the PDF parameters. The goal is to treat $\alpha _s$ on the same footing as the vector of parameters $\theta$ that determine the PDFs, i.e. to simultaneously minimize the figure of merit with respect to both $\alpha _s$ and $\theta$. This is difficult in practice, because the dependence on $\alpha _s$ appears in the theoretical predictions, which, for reasons of computational efficiency, are provided in the form of pre-computed grids determined before the fit using the APFELgrid framework [31, 32].

This difficulty can be overcome through the correlated replica method, as we now explain. The method relies on the concept of “correlated replica”, or c-replica for short. A c-replica is a correlated set of PDF replicas, all obtained by determining the best-fit $\theta ^{(k)}$ Eq. (2.4) but with different (fixed) values of $\alpha _s$: given the data replica $D^{(k)}$, the minimization Eq. (2.4) is performed several times, for a range of fixed values of $\alpha _s(m_Z)$. A c-replica thus corresponds to as many standard NNPDF replicas as the number of values of $\alpha _s$ for which the minimization has been performed, all obtained by fitting to the same underlying data replica $D^{(k)}$.

One can then determine the best-fit value $\alpha _s^{(k)}$ for the *k*th c-replica by minimizing as a function of $\alpha _s$ the figure of merit $\chi ^2$ Eq. (2.3) computed with $\theta ^{(k)}(\alpha _s)$ as $\alpha _s$ is varied for fixed *k*. Namely, we first define the figure of merit computed for the *k*th c-replica,

2.5 $$\begin{aligned} \chi ^{2(k)}(\alpha _s)=\chi ^{2} \left( \alpha _s, \theta ^{(k)}(\alpha _s),D^{(k)} \right) , \end{aligned}$$

which we can view as a function of $\alpha _s$. Note that the dependence of the theory prediction *T* and thus of the figure of merit Eq. (2.3) on $\alpha _s$ is both explicit, and implicit through the best-fit parameters $\theta ^{(k)}(\alpha _s)$. We then determine the best-fit value of $\alpha _s$ for the *k*th c-replica as

2.6 $$\begin{aligned} \alpha _s^{(k)}=\mathrm {argmin}\left[ \chi ^{2(k)}(\alpha _s)\right] . \end{aligned}$$

Note that while, as discussed above, in order to avoid overfitting, the best-fit $\theta ^{(k)}$ is not the absolute minimum of the figure of merit, no overfitting of $\alpha _s$ is possible, because overfitting happens when fitting a function, not a single parameter. Hence, in Eq. (2.6) the best fit value $\alpha _s^{(k)}$ does denote the absolute minimum. Therefore, in practice $\alpha _s^{(k)}$ can be determined by fitting a parabola to the discrete set of values of $\chi ^2(\alpha _s)$ for each replica, and finding the minimum of the parabola.

Note also that determining the best-fit for the *k*th c-replica by first minimizing with respect to $\theta$ and then minimizing with respect to $\alpha _s$ is equivalent to simultaneously minimizing in the $(\alpha _s,\theta )$ hyperspace, provided the same figure of merit is used for PDF and $\alpha _s$ determination. For instance, the absolute minimum in $(\alpha _s,\theta )$ is the solution to the coupled equations

2.7 $$\begin{aligned} \frac{\partial }{\partial \theta }\chi ^2(\alpha _s,\theta )= & {} 0, \end{aligned}$$

2.8 $$\begin{aligned} \frac{\partial }{\partial \alpha _s}\chi ^{2}(\alpha _s,\theta )= & {} 0, \end{aligned}$$

where Eq. (2.7) is actually a system of $N_\mathrm{par}$ equations because $\theta$ is an $N_\mathrm{par}$-component vector and the partial derivative is a gradient. On the other hand, this solution can also be found (compare Fig. 1) by first finding the solution $\theta (\alpha _s)$ to Eq. (2.7), determining $\chi ^2(\alpha _s) = \chi ^{2}(\alpha _s,\theta (\alpha _s) )$, and solving

2.9 $$\begin{aligned} \frac{d}{d\alpha _s}\chi ^{2}(\alpha _s) = \left( \frac{\partial }{\partial \alpha _s}+\frac{\partial \theta }{\partial \alpha _s} \frac{\partial }{\partial \theta } \right) \chi ^{2}(\alpha _s,\theta )=0. \end{aligned}$$

This two stage procedure yields the same solution as the coupled Eqs. (2.7)–(2.8) because the second term in brackets on the r.h.s. of Eq. (2.9) vanishes since $\theta (\alpha _s)$ was the solution of Eq. (2.7).

One thus ends up, for each data replica $D^{(k)}$, with a best fit value $(\alpha _s^{(k)},\theta ^{(k)})$ of both $\alpha _s$ and the PDF parameters. That is, from each c-replica we extract a single best fit value $\alpha _s^{(k)}$ – an “$\alpha _s$ replica” – exactly on the same footing as all the other fit parameters. The ensemble of values $\alpha _s^{(k)}$ obtained from all the c-replicas then provides a representation of the probability density of $\alpha _s$ from which we can perform statistics in the usual way. Interestingly, this means that we can now not only compute the best fit $\alpha _s$ and its uncertainty as the mean and standard deviation (or indeed 68% confidence interval) using the $\alpha _s$ replicas, but also the correlation between $\alpha _s$ and the PDFs or indeed any PDF-dependent quantity.

In summary, the correlated replica method is akin to the standard NNPDF methodology in that it starts by producing a set of replicas of the original data, but uses these to construct a set of correlated $\alpha _s$-dependent PDF replicas, the c-replicas, which correspond to parameters $\theta ^{(k)}(\alpha _s)$ when *k* runs over the replica sample and $\alpha _s$ takes a number of discrete values. From each c-replica a best-fit $\alpha _s^{(k)}$ can then be determined, so each c-replica yields an $\alpha _s$ replica, with $\alpha _s^{(k)}$ defined by Eq. (2.6).

Hence, the correlated replica method exploits the fact that in the NNPDF approach it is sufficient to know the best-fit set of parameters for each replica, and all other information is contained in the replica sample. The price to pay for this is that the statistics of the $\alpha _s$ fitting is inevitably more demanding than with the method of Refs. [17, 18] because we have now have to fit a different parabola for each c-replica. The issues arising from this will be discussed in the next section.

### Implementation

Building on the conceptual strategy described above, we now present the practical implementation of the correlated replica method. As already mentioned, the best-fit $\alpha _s^{(k)}$ Eq. (2.6) for the *k*th c-replica is determined by fitting a parabola to the figure of merit $\chi ^2(\alpha _s)$, viewed as a function of $\alpha _s$, known at the discrete set of $\alpha _s$ values for which best-fit $\theta ^{(k)}(\alpha _s)$ are available. The reliability of the quadratic approximation to $\chi ^{2(k)}$ Eq. (2.5) and the stability of the position of the minimum upon inclusion of higher order terms can be studied using standard methods and will be discussed in Sect. 3.2 below.

The best-fit $\alpha _s$ and its uncertainty are then determined, according to standard NNPDF methodology, as the mean and standard deviation computed over the sample of $\alpha _s$ replicas

2.10 $$\begin{aligned} \alpha _s= \langle \alpha _s^{(k)}\rangle _\mathrm{rep};\quad \sigma _\alpha =\mathrm {std} \left( \alpha _s^{(k)}\right) _\mathrm{rep}, \end{aligned}$$

where $\alpha _s^{(k)}$ is given by Eq. (2.6).

The uncertainty due to the finite size of the replica sample can be estimated by bootstrapping. To this purpose, one constructs $N_\mathrm{res}$ resamples of the original sample of $N_\mathrm{rep}$ values $\alpha _s^{(k)}$. Each resample is obtained by drawing at random $N_\mathrm{rep}$ values from the original sample by allowing repetition. This means that each resample differs from the original sample because some values are repeated and others are missing. The finite-size uncertainty is then estimated by first computing the mean $\alpha _s^{(\mathrm{res}, i)}$ for each of the resamples,

2.11 $$\begin{aligned} \alpha _s^{(\mathrm{res}, i)}=\langle \alpha _s\rangle _\mathrm{rep}, \end{aligned}$$

where the mean is computed over the $N_\mathrm{rep}$ values of the *i*th resample. The bootstrapping estimate of the finite-size uncertainty on the central value of $\alpha _s$ is then the standard deviation of the set of $\alpha _s^{(\mathrm{res}, i)}$

2.12 $$\begin{aligned} \Delta _{\alpha _s} = \mathrm {std} \left( \alpha _s^{(\mathrm{res}, i)}\right) _\mathrm{res}. \end{aligned}$$

The uncertainty on the uncertainty $\Delta _{\sigma }$ can be similarly computed by first determining the uncertainty Eq. (2.10) for each resample, thus leading to an uncertainty $\sigma _\alpha ^{(\mathrm{res}, i)}$, and then computing the standard deviation of the ensuing uncertainties:

2.13 $$\begin{aligned} \Delta _{\sigma } = \mathrm {std} \left( \sigma _\alpha ^{(\mathrm{res}, i)}\right) _\mathrm{res}. \end{aligned}$$

We find that results become independent of the random seed used to generate the bootstrapping resamples when $N_\mathrm {res}\simeq 10{,}000$.

It turns out that, when determining the best-fit $\theta ^{(k)}(\alpha _s)$ through the standard NNPDF minimization algorithm, a certain amount of fluctuation of individual values of $\chi ^2(\alpha _s)$ about the parabolic best-fit is observed. In other words, the $\chi ^2$ profiles as a function $\alpha _s$ are not very smooth. It is therefore advantageous to introduce an improvement of the algorithm, called batch minimization, which increases its accuracy at the cost of increasing the time required for fitting.

Furthermore, when using the standard NNPDF minimization, occasionally the fit fails to satisfy a number of convergence and quality criteria (see Sect. 3.3.2 of Ref. [26]), in which case it is discarded. Consequently, for some c-replicas $\chi ^2(\alpha _s)$ is not available for all $\alpha _s$ values. One must then decide on a sensible criterion for c-replica selection, with the most restrictive criterion being to only accept c-replicas for which all $\chi ^2(\alpha _s)$ values are available, and the least restrictive one to accept c-replicas for which at least three $\chi ^2(\alpha _s)$ values are available so a parabola can be fitted. We now discuss batch minimization and replica selection criteria in turn.

The idea of batch minimization is to refit a given set of data replicas more than once. In order to improve the smoothness of the $\chi ^2$ profiles obtained by the direct use of NNPDF minimization, we exploit the fact that the minimization algorithm is not deterministic, and thus simply rerunning the minimization from a different random seed leads to a slightly different answer. Each of these refits is called a batch. For each c-replica *k* and each $\alpha _s$ value we then end up with several best-fit results $\theta ^{(k)}_i(\alpha _s)$, where *i* runs over batches.

We then pick for each c-replica *k* and for each $\alpha _s$ value the batch which gives the best $\chi ^2$. We also impose the condition that at least two of the batches for the given c-replica and $\alpha _s$ value have converged, in order to mitigate the influence of outliers that narrowly pass the post-selection fit criteria. The dependence of results on the number of batches used can then be assessed a posteriori by comparing results found with different numbers of batches.

After batch minimization, we end up with a set of c-replicas $\theta ^{(k)}(\alpha _s)$ where, however, for several c-replicas, results may be missing for one or more $\alpha _s$ values because convergence was not achieved. We must thus determine the minimum number of $\alpha _s$ values $N_\mathrm {min}$ such that a c-replica is accepted. The threshold $N_\mathrm {min}$ is chosen to ensure the stability of results. Curves with too few points lead to an unreliable parabolic fit, and thus an unreliable best-fit $\alpha _s^{(k)}$ for that c-replica. This then leads to outlier values of $\alpha _s^{(k)}$ and a spuriously large value of the uncertainty on the $\alpha _s^{(k)}$ determination. On the other hand, once the number of points is sufficient for a reliable parabolic fit, requiring more points does not improve the determination of $\alpha _s^{(k)}$, but it reduces the number of c-replicas which are retained in the final sample, which in turn increases the finite-size uncertainty.

Therefore, the optimal value of $N_\mathrm {min}$ arises from a trade-off between the uncertainty on $\alpha _s^{(k)}$ from the parabolic fitting, and the finite-size uncertainty. In order to keep both criteria into account, we fix $N_\mathrm {min}$ by minimizing the bootstrapping uncertainty $\Delta _{\sigma }$ Eq. (2.13). However, in order to make sure that the selection is not too tight, we do not minimize $\Delta _{\sigma }$ itself. Rather, we first multiply it by a penalty factor that depends on the number of points. This is in turn determined as the 99% confidence level factor from a two sided Student *t* distribution. Indeed, if the distribution of best-fit $\alpha _s^{(k)}$ over replicas is Gaussian, then the difference between the sampled and true central value follows a Student *t* distribution with $N_\mathrm {rep}-1$ degrees of freedom, zero mean and scale parameter $\Delta _{\sigma }/\sqrt{N_\mathrm {rep}}$. A given confidence level around the mean is equal to the standard deviation $\Delta _{\sigma } T_{\mathrm{CL},(N_\mathrm {rep}-1)}$, where $T_{\mathrm{CL},N}$ is the percentile at CL confidence level for the two-sided confidence factor of the Student *t* distribution with *N* degrees of freedom. Hence, we choose a $99\%$ confidence level, and we determine $N_\mathrm {min }$ as the value yielding the minimum of $\Delta _{\sigma } T_{0.99,(N_\mathrm {rep}-1)}$. Also in this case, the dependence of results on the choice of selection criteria can be studied a posteriori, and will be discussed in Sect. 3.2.

## The strong coupling constant from NNPDF3.1

We now present the main result of this work, namely the determination of $\alpha _s\left( m_Z \right)$ based on the methodology discussed in Sect. 2. We first present the best-fit result for $\alpha _s$ and its experimental uncertainty, determined through the correlated replica method. We then discuss methodological and theoretical uncertainties. We finally collect our final result and briefly compare it to other recent determinations from PDF fits and to the PDG average.

### Best-fit results for $\alpha _s$ and statistical uncertainty

We have determined $\alpha _s\left( m_Z \right)$ both at NLO and NNLO using the methodology and dataset of the NNPDF3.1 global analysis [16]. The only difference in the fit settings is the theoretical description of the inclusive jet production datasets at NNLO. Here we use exact NNLO theory [33] for the ATLAS [34] and CMS [35] inclusive jet measurements at 7 TeV, and discard the other jet datasets used in NNPDF3.1 for which the NNLO calculation is not available (note that, as in NNPDF3.1, only ATLAS data in the central rapidity bin are included). To ensure a consistent comparison, the input datasets of the NLO and NNLO fits are identical, up to small differences in the kinematical cuts as explained in [16].

Specifically, we determine $\alpha _s$ by generating a set of 400 data replicas, and from them a set of 400 c-replicas each with 21 values of $\alpha _s$, thus corresponding to a total of 8400 PDF replicas correlated as discussed in Sect. 2.1. These c-replicas are generated for $\alpha _s\left( m_Z \right)$ ranging between 0.106 and 0.130, varied in steps of $\Delta _{\alpha _s}=0.002$ between 0.106 and 0.112 and between 0.128 and 0.130, and in steps of $\Delta _{\alpha _s}=0.001$ between 0.112 and 0.128, adding up to the total of 21 values. From these we determine $\alpha _s$ replicas, which form a representation of the probability distribution of $\alpha _s$.

At NNLO we find

3.1 $$\begin{aligned} \alpha _s^\text {NNLO}(m_Z) = 0.11845 \pm 0.00052~(0.4\%). \end{aligned}$$

This result is based on a total of $N_\mathrm{rep}=379$ c-replicas, selected from a starting set of 400 after batch minimization of three batches, using the minimization and selection methods described in Sect. 2.2. At NLO we find

3.2 $$\begin{aligned} \alpha _s^\text {NLO}(m_Z) = 0.12067 \pm 0.00064~(0.5\%) . \end{aligned}$$

In this case, the sample includes $N_\mathrm{rep}=108$ c-replicas selected after batch minimization with two batches. The smaller number of c-replicas selected at NLO is in part explained by the requirement (see Sect. 2.2) that two batches have converged for the given $\alpha _s$ value, which is of course less severe when three batches are available, but the worse quality of the NLO fit also plays a role since it causes more fits to be discarded by the post-selection criteria.

The uncertainty quoted in Eqs. (3.1) and (3.2) is that obtained using standard NNPDF methodology, namely, taking the standard deviation over the $\alpha _s$ replica sample. We have verified that essentially the same results are obtained if instead we compute the 68% confidence interval. The uncertainty is obtained in precisely the same way as our PDF uncertainty, to which it is strongly correlated; it includes the propagated correlated uncertainty from the underlying data, and uncertainties coming from possible inefficiencies of the minimization procedure. This uncertainty is what we refer to as the experimental uncertainty on $\alpha _s\left( m_Z \right)$. It will have to be supplemented by methodological and theoretical uncertainties, to be discussed in Sects. 3.2 and 3.3 below.

**Fig. 2:**
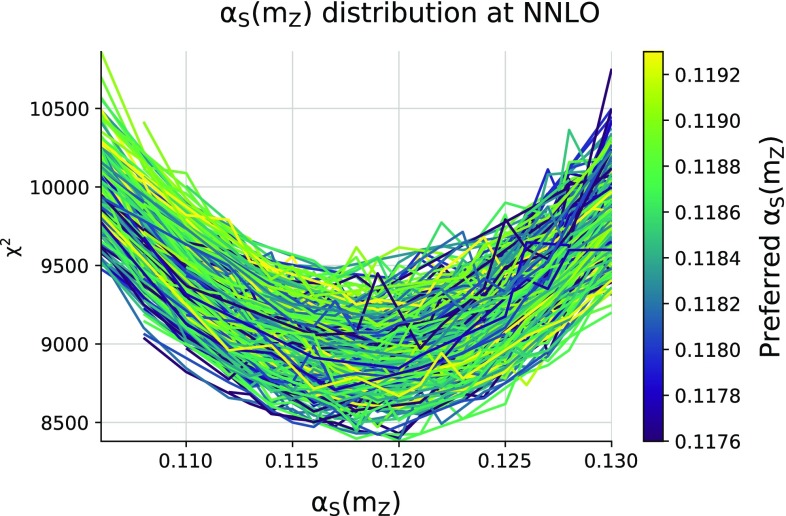
The $\chi ^2$ profiles for each of the 379 c-replicas used for the NNLO determination of $\alpha _s(m_Z)$, Eq. (3.1). Each curve corresponds to an individual c-replica, and the color scale indicates the best-fit $\alpha _s$ value determined from the parabolic fit to that curve

**Fig. 3:**
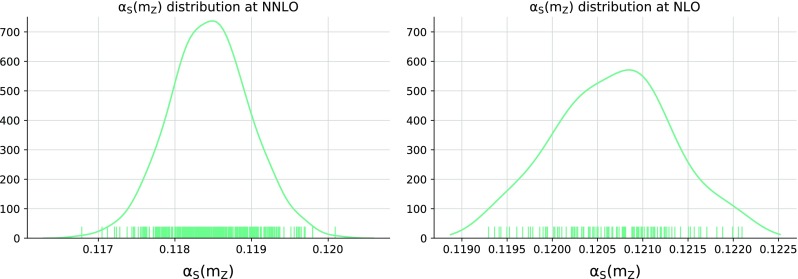
The probability distributions for the best-fit $\alpha _s^{(k)}$ values Eq. (2.6) at NNLO (left) and at NLO (right). Each marker indicates the $\alpha _s^{(k)}$ value corresponding to each individual c-replica

The 379 c-replicas selected for the NNLO determination are shown in Fig. 2. The color scale of each curve indicates the best-fit $\alpha _s$ value. It is apparent that the vast majority of the curves exhibit an approximately parabolic behaviour. The probability distributions of the best-fit values $\alpha _s^{(k)}$ Eq. (2.6) which correspond to each c-replica, both at NLO and at NNLO, are shown in Fig. 3, where the markers indicate the value of $\alpha _s^{(k)}$ for each specific c-replica. These probability densities have been determined using the Kernel Density Estimate method, see [36]. We find that the probability distribution for $\alpha _s\left( m_Z \right)$ is both shifted to higher values and broadened when going from NNLO to NLO. The decrease of the best-fit value of $\alpha _s\left( m_Z \right)$ when going from NLO to NNLO has been repeatedly observed before (see Table 1 of Ref. [37] for an extensive set of examples), also in our previous determination [17, 18], while the broadening is due to the poorer quality of the NLO fit.

The impact on the $\alpha _s$ determination of any subset of the input data can be roughly assessed by studying its contribution to the total figure of merit. We have done this by determining replica by replica the corresponding partial $\chi ^2_p$ for a process (or group of processes) *p*, defined as the figure of merit Eq. (2.3) with the summation over *i*, *j* now restricted to data which belong to the specific subset *p*. The $\alpha _s$ fit procedure through the correlated replica method is then just repeated but using this partial $\chi ^2_p$. Namely, for each c-replica the partial $\chi ^{2(k)}_p$ for process *p* is computed, a parabola is fitted to it, the corresponding minimum $\alpha _{s,p}^{(k)}$ of the parabola is determined, and the resulting set of minima is used to find the value of $\alpha _s\left( m_Z \right)$ and its uncertainty.

**Table 1:** Number of data points at NLO and NNLO corresponding to the different subsets of the input experimental data considered here. These eight subsets add up to the total dataset

	NLO	NNLO
Fixed-target charged lepton DIS	973	973
Fixed-target neutrino DIS	908	908
Collider DIS (HERA)	1221	1211
Fixed Target Drell–Yan	189	189
Collider Drell–Yan	378	388
Inclusive jets	164	164
*Z* $p_T$	120	120
Top quark pair production	26	26
Total	3979	3979

Here we consider the following eight groups of processes *p*: top production, the *Z* $p_T$ distributions, collider and fixed target Drell–Yan, inclusive jets, and deep-inelastic scattering (DIS) either at HERA or at fixed-target experiments, in the latter case separating charged lepton and neutrino beams. The number of data points corresponding to each of these data subsets is shown in Table 1. Not unexpectedly, the $\chi ^{2(k)}_p$ profiles for data subsets turn out to be rather less parabolic than the total $\chi ^2$, especially for processes such as neutrino DIS or fixed target Drell–Yan that have weak sensitivity to $\alpha _s$.

**Fig. 4:**
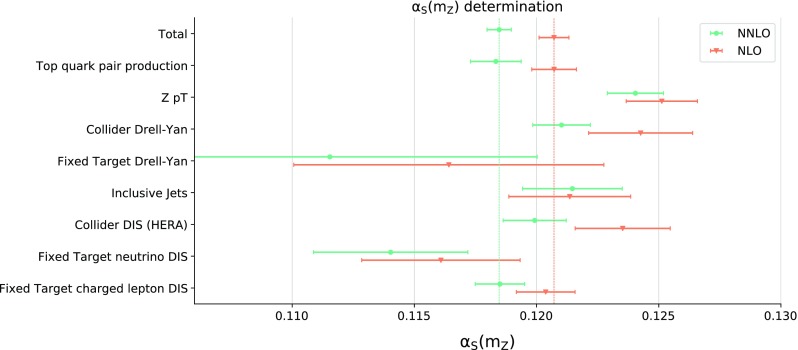
The values of the partial $\alpha _s\left( m_Z \right)$ and the corresponding uncertainties determined from $\chi ^{2}_p$ for the various families of processes *p* of Table 1 at NLO and NNLO

When determining $\alpha _s\left( m_Z \right)$ from the partial $\chi ^{2(k)}_p$, we do not repeat the replica selection and simply use the same replicas selected for the total dataset. Consequently, we must apply a form of post-selection, whereby each time a parabola for $\chi ^{2(k)}_p$ has no minimum the corresponding c-replica is ignored. At NNLO, for five out of eight data subsets we retain all 379 c-replicas, while for jets, neutrino DIS, and fixed-target Drell–Yan, we retain only 376, 366, and 302 c-replicas respectively. The results for the partial $\alpha _s\left( m_Z \right)$ determined from $\chi ^{2}_p$ for the various families of processes are collected in Fig. 4. The central value and uncertainty shown are respectively determined as the median and 68% symmetric confidence level interval from the corresponding partial $\alpha _{s,p}^{(k)}$. This is because the analogue of Fig. 3 for individual processes turns out to be rather non-gaussian, especially for processes such as fixed-target Drell–Yan that only have a weak handle on $\alpha _s$.

The values of $\alpha _s\left( m_Z \right)$ shown in Fig. 4 should be interpreted with some care. Indeed, the partial $\chi ^2_p$ is in each case computed using PDF c-replicas determined from the minimization of the global $\chi ^2$. These are in general different from the c-replicas that would be determined by simultaneous minimization of $\chi ^2_p$ with respect to $\alpha _s$ and the PDFs. Therefore, the values of $\alpha _{s,p}$ in Fig. 4 cannot be interpreted as the best-fit values of $\alpha _s\left( m_Z \right)$ for a given subset *p*. They instead provide an estimate of the pull on the best-fit $\alpha _s\left( m_Z \right)$ value that specific families of processes have within the global fit subject to the constraints from the rest of the data.

Moreover, even their interpretation as pulls is only approximate. Firstly, the replica selection is applied to the total $\chi ^2$ rather than to each partial $\chi ^2_p$, so that several partial $\chi ^{2(k)}_p$ profiles turn out not to have a minimum. Furthermore, the total $\chi ^2$ includes cross-correlations which are lost when determining partial $\chi ^2_p$, because the covariance matrix $C_{t_0}$ in Eq. (2.3) is generally nonzero even when *i* and *j* belong to different data subsets. For instance, inclusive jet, *Z* $p_T$, and Drell–Yan measurements from the same experiment (ATLAS, or CMS) are correlated amongst themselves by the common luminosity uncertainty. Finally, partial $\alpha _s$ values are correlated through the underlying PDFs, implying that the pulls should not be expected to combine additively into the final result.

Even with all these caveats, Fig. 4 shows that the very accurate $\alpha _s\left( m_Z \right)$ value from the global dataset is obtained from a combination of pulls which correspond to values of $\alpha _s\left( m_Z \right)$ dispersed about the global best-fit value, without signs of tension or inconsistency, and subject to significant fluctuations which are suppressed when constructing the total $\chi ^2$. This supports our conclusion that the current determination of $\alpha _s\left( m_Z \right)$ from a global fit is more precise and accurate than determinations based on subsets of data relying on pre-existing PDF sets.

**Fig. 5:**
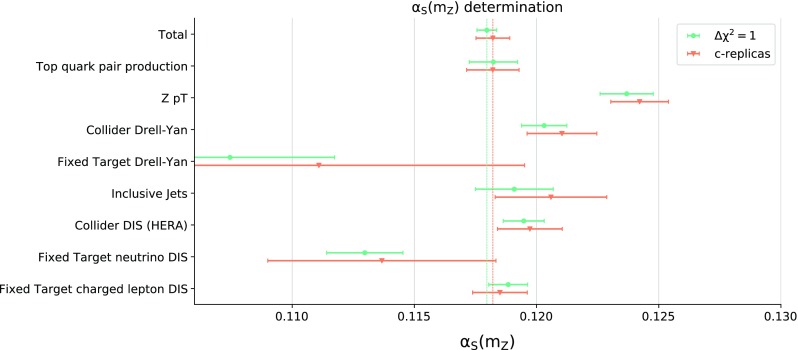
Comparison of the NNLO determination of $\alpha _s\left( m_Z \right)$ using the method of [17, 18], which neglects the correlation between $\alpha _s$ and PDFs, and the current one based on the correlated replicas

**Fig. 6:**
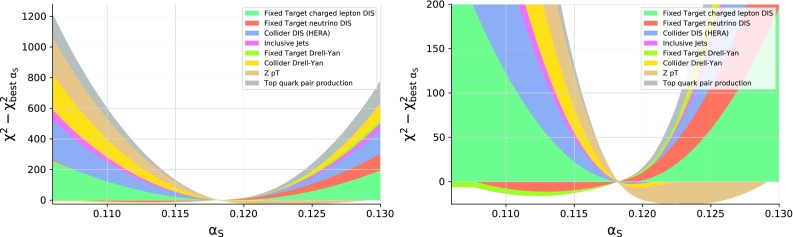
The NNLO cumulative differences, $\chi ^2_p(\alpha _s)-\chi ^2_p(0.1185)$, between the partial $\chi ^2_p$ values evaluated at $\alpha _s\left( m_Z \right)$ and at best-fit value $\alpha _s\left( m_Z \right) =0.1185$ for different families of processes. In the part of the plot above (below) zero, only contributions from experiments for which the cumulative difference is positive (negative) are shown (see text). The plot is displayed either with a wider (left) or narrower (right) choice of range on the *y* axis

Finally, we compare the current NNLO determination of $\alpha _s\left( m_Z \right)$, Eq. (3.1) and Fig. 4, with the one found using the method of Refs. [17, 18]. We fix $\alpha _s$ and add the contribution to the $\chi ^2$ from each standard PDF replica for that $\alpha _s$ value. We then determine the total $\chi ^2(\alpha _s)$, fit a parabola to it, and determine the best-fit and uncertainty as the minimum and $\Delta \chi ^2=1$ interval. For simplicity, we do this without using batch minimization, i.e. we compute the total $\chi ^2$ from one of the batches (batch II, see Sect. 3.2 below) which then enter the batch minimization procedure. Using this method we find

3.3 $$\begin{aligned} \alpha _s^\text {NNLO}(m_Z)= & {} 0.1180 \pm 0.0004~(0.3\%), \nonumber \\ \alpha _s^\text {NLO}(m_Z)= & {} 0.1203 \pm 0.0004~(0.3\%). \end{aligned}$$

Also in this case we can repeat the determination for different data subsets based on the partial $\chi ^2_p$, and the corresponding results are compared in Fig. 5.

As expected, and discussed in the introduction and in Sect. 2.1, we find that the best-fit values of $\alpha _s\left( m_Z \right)$ determined with the old method [17, 18] and with the new correlated replica method are in good agreement, both for the global dataset and for the data subsets. The small differences in central values are most likely due to uncertainties related to the finite size of the replica sample, which, as discussed in [17, 18], can be non-negligible when the old method is used. On the other hand, also as expected, neglecting the correlation between $\alpha _s$ and PDFs as in the old method leads in general to an underestimate of the uncertainty on $\alpha _s$. This effect is more marked for processes such as fixed-target Drell–Yan and neutrino DIS that have a limited sensitivity to $\alpha _s$, because in this case the difference in length of the semi-axes of the error ellipse in Fig. 1 is large.

This determination of $\alpha _s\left( m_Z \right)$ from the total $\chi ^2$ also offers a complementary way of quantifying how much each family of processes constrains the final best-fit value, by plotting the contribution of each data subset to the total $\chi ^2$. Specifically, we show in Fig. 6 the cumulative differences at NNLO, $\chi ^2_p(\alpha _s)-\chi ^2_p(0.1185)$, between each partial $\chi ^2_p$ and its value computed at the global best-fit $\alpha _s\left( m_Z \right)$ value, neglecting cross-correlations between different data subsets. The plot is divided into two halfs: above zero, only positive differences are shown, and below zero, only negative ones. Thus, when all differences are positive the plot shows the breakdown of the total $\chi ^2$ into the contribution of different experiments (up to neglected cross-correlations), while when some of them are negative the lower part of the plot shows by how much the $\chi ^2$ of the individual experiments shown has improved in comparison to their value at the global minimum $\alpha _s(M_z)=0.1185)$. In order to increase readability, the plot is displayed twice, with two different choices of scale on the *y* axis.

From this comparison, we observe that the LHC data significantly contribute to constraining $\alpha _s$. In particular, it is interesting to note that the 13 data points from top-quark pair production lead to a significant contribution to the total $\chi ^2$ away from the best-fit, even though the global dataset contains almost 4000 data points. Similar considerations apply to the *Z* $p_T$ distributions. This means that there is a small range of values of $\alpha _s$ where these two groups of processes are consistent with the rest of the data entering the fit, thereby providing a tight constraint on $\alpha _s$.

### Methodological uncertainties

In view of the rather small experimental uncertainty on the final value of $\alpha _s\left( m_Z \right)$, Eqs. (3.1)–(3.2), we need to assess possible uncertainties associated to the various aspects of our methodology described in Sect. 2. Specifically, we discuss here the methodological uncertainties associated to c-replica selection, batch minimization, the quadratic approximation to $\chi ^2$ profiles, and the treatment of correlated systematics.

The replica selection algorithm determines an optimal value of $N_\text {min}$, the minimal number of $\alpha _s$ for which results must be available for a given c-replica to be selected. We have varied this value from its minimum $N_\text {min}=3$ (needed in order to fit a parabola) to a high value $N_\text {min}=18$ (meaning that at most three values $\alpha _s$ can be missing in order for a c-replica to be retained). Results for the number of c-replicas passing the criterion and the ensuing value of $\alpha _s$ are collected in Table 2 for a number of choices. In each case we also show the finite-size uncertainty $\Delta _{\alpha _s}$ on the best-fit $\alpha _s$ estimated by bootstrapping, Eq. (2.12).

The number of surviving c-replicas varies significantly; all the starting 400 c-replicas pass the loosest criterion (i.e., it is always possible to fit a parabola to any c-replica), but only $N_\text {rep}=12$ c-replicas pass the most restrictive criterion. However, even with this most restrictive criterion the finite-size uncertainty is below the permille level. For the value selected by the algorithm, the finite-size uncertainty is of order $0.03\%$, i.e. by almost a factor 20 smaller than the experimental uncertainty Eq. (3.1) and it does not decrease further even when all c-replicas are kept. The finite-size uncertainty on the $\alpha _s$ uncertainty $\Delta _{\sigma }$ itself Eq. (2.13) is comparable in all cases.

The value of $\alpha _s\left( m_Z \right)$ and its experimental uncertainty are hence very stable; the shift of central value and uncertainty when the selection criterion is varied is always smaller than the finite-size uncertainty. This stability can be understood by observing that each c-replica consists of at least $N_\mathrm{min}$ correlated PDF replicas, so each of the determinations shown in Table 2 is obtained from more than $N_\mathrm{min}\times N_\mathrm{rep}$ PDF replicas. We thus estimate that the bootstrapping uncertainty, and the related but smaller uncertainty due to choice of replica selection, to be of order $\Delta _{\alpha _s}=0.00003~(0.03\%)$, one order of magnitude smaller than the experimental uncertainty.

**Table 2:** Dependence of the NNLO determination of $\alpha _s\left( m_Z \right)$ on the minimum number of $\alpha _s$ values per c-replica $N_\mathrm{min}$ (see Sect. 2.2). In each case, the best fit value and statistical uncertainty on $\alpha _s$ are shown, together with the number of surviving c-replicas $N_\mathrm{rep}$ and the bootstrapping uncertainty $\Delta _{\alpha _s}$ Eq. (2.12). The value chosen using the selection criterion of Sect. 2.2, which leads to the final vale of $\alpha _s\left( m_Z \right)$ Eq. (3.1), is $N_\text {min}=6$ (third row of the table, in boldface)

$N_\text {min}$	$\alpha _s\left( m_Z \right)$	$N_\mathrm{rep}$	$\Delta _{\alpha _s}$
18	$0.11842\pm 0.00031~(0.3\%)$	12	0.00009
15	$0.11844\pm 0.00044~(0.4\%)$	92	0.00005
**6**	$\mathbf{0.11845\pm 0.00052~(0.5\%)}$	**379**	**0.00003**
3	$0.11844 \pm 0.00056~(0.5\%)$	400	0.00003

**Table 3:** Results for the NNLO determinations of $\alpha _s\left( m_Z \right)$ using different combinations of the three available batches. In each case we show both the best-fit value of $\alpha _s\left( m_Z \right)$, the minimum number of $\alpha _s$ values per c-replica $N_\mathrm{min}$, and the corresponding number surviving c-replicas $N_\mathrm{rep}$. The last row (in boldface) corresponds to our final result Eq. (3.1)

Batches	$\alpha _s\left( m_Z \right)$	$N_\mathrm{min}$	$N_\mathrm{rep}$
I	$0.11831\pm 0.00065~(0.5\%)$	9	310
II	$0.11828\pm 0.00062~(0.5\%)$	14	216
III	$0.11822 \pm 0.00072~(0.6\%)$	13	369
I + II	$0.11844 \pm 0.00054~(0.5\%)$	11	225
I + III	$0.11841 \pm 0.00058~(0.5\%)$	13	158
II + II	$0.11841 \pm 0.00060~(0.5\%)$	14	288
**I + II + III**	$\mathbf{0.11845 \pm 0.00052~(0.4\%)}$	**6**	**379**

We next turn to discuss batch minimization. The results shown in Table 2 all correspond to the NNLO baseline which uses batch minimization with three batches. In order to assess the impact of batch minimization, in Table 3 we compare results obtained with each of the three batches, with the three possible pairs, and combining the three batches. In each case we show the final best-fit $\alpha _s\left( m_Z \right)$ and experimental uncertainty, the value of $N_\mathrm{min}$, the minimum number of $\alpha _s^{(k)}$ values per c-replica, and the number of surviving c-replicas $N_\mathrm{rep}$.

It is clear from this comparison that as more batches are combined, results become more stable. The values of $N_\mathrm{min}$ are on average larger with two batches, and larger still with three, but without a reduction of the number of surviving c-replicas $N_\mathrm{rep}$ as was observed in Table 2. With three batches, $N_\mathrm{rep}$ is largest even though $N_\mathrm{min}$ is also largest. This means that, thanks to batch minimization, the number of available $\alpha _s^{(k)}$ values per replica is on average higher. It follows that the finite-size uncertainty is reduced by batch minimization, thus leading to the very small uncertainties shown in Table 2.

The values of $\alpha _s\left( m_Z \right)$ behave as expected upon use of batch minimization. The experimental uncertainty is reduced when more batches are used and the central values with different combinations of batches are all consistent with each other within given uncertainties. Furthermore, the differences in central values with different combinations of batches are reduced upon use of batch minimization (they are smaller when using two batches than when using a single batch). Additionally, the shift in central value when increasing the number of batches is rather smaller than the uncertainty, and, finally, the central value is stabilized when increasing the number of batches, so the difference between two and three batches is on average smaller than the difference between one and two batches.

**Table 4:** Results for the NNLO determinations of $\alpha _s\left( m_Z \right)$ when the $N_\mathrm{trim}$ outer values of $\alpha _s$ are not used and the fit is restricted to a smaller range. In the bottom part of the table we also show results found discarding values asymmetrically, at the upper or lower edge of the range. In each case we show the number of discarded $\alpha _s$ values, the best-fit value of $\alpha _s\left( m_Z \right)$, and the number of surviving c-replicas $N_\mathrm{rep}$. The first row (in boldface) corresponds to our final result Eq. (3.1)

$N_\mathrm{trim}$	Fitted $\alpha _s\left( m_Z \right)$ range	$\alpha _s\left( m_Z \right)$	$N_\mathrm{rep}$
**0**	$\mathbf{[0.106,0.130]}$	$\mathbf{0.11845\pm 0.00052~(0.4\%)}$	**379**
2	[0.108, 0.128]	$0.11846 \pm 0.00045~(0.4\%)$	218
5	[0.110, 0.126]	$0.11852\pm 0.00051~(0.4\%)$	290
10	[0.114, 0.124]	$0.11869 \pm 0.00046~(0.4\%)$	32
15	[0.115, 0.120]	$0.11822 \pm 0.00079~(0.7\%)$	10
4	[0.113, 0.130]	$0.11850 \pm 0.00058~(0.5\%)$	296
5	[0.106, 0.124]	$0.11855 \pm 0.00059~(0.5\%)$	197

We conclude that the value of $\alpha _s\left( m_Z \right)$ found using three batches is the most accurate. We observe that even the shift between the three-batch value and the single-batch value which differs most from it is about a third of the finite-size uncertainty. We take this as further evidence that there is no extra contribution of methodological origin due to batch minimization to be added to the statistical uncertainty. We finally observe that the two-batch result is in fact consistent within its very slightly larger uncertainty, thus justifying the use of only two batches at NLO.

We next turn to the methodological uncertainties related to the quadratic fitting of $\chi ^2$ profiles. We have studied this in three different ways: by removing outer values of $\alpha _s\left( m_Z \right)$ from the fit; by adding higher order terms to the fitting function; and by changing the fitting variable. We discuss each in turn.

First, we have repeated the NNLO determination removing $\alpha _s$ values that are farthest from the best-fit value $\alpha _s\left( m_Z \right) =0.1185$, fitting a smaller range of values around the minimum. As a further consistency check, we have removed $\alpha _s$ values asymmetrically. Results are shown in Table 4; in each case we show the number of discarded $\alpha _s$ values $N_\mathrm{trim}$, the resulting fitted range, the best fit $\alpha _s\left( m_Z \right)$ and uncertainty, and the number of surviving c-replicas $N_\mathrm{rep}$. Here too, the behaviour is consistent with expectations. As the fitted range is reduced, the experimental uncertainty increases and the number of surviving c-replicas decreases (thereby also increasing the finite-size uncertainty). The central value, however, is extremely stable; the shift in central value when restricting the range is always more than a factor two smaller than the experimental uncertainty. In fact, the shift is never larger than $\Delta =0.00010~(0.08\%)$ unless the number of surviving c-replicas becomes of order ten, in which case the finite-size uncertainty (recall Table 2) is of the same order or larger.

A different way of testing for deviations from quadratic behaviour is to apply a criterion to assess fit quality to both quadratic and cubic fits. Here we use the Akaike Information Criterion (AIC) [38], which estimates the expected relative distance between a given fitted model and the unknown underlying law [39]. The AIC score balances goodness of fit against simplicity of the model. A lower score corresponds to a lower expected distance measured by the Kullback–Leibler divergence. The AIC score is defined by

3.4 $$\begin{aligned} \mathrm {AIC} = 2r - 2\ln {L} + \frac{2r(r+1)}{n-r-1}, \end{aligned}$$

where *r* is the number of degrees of freedom of the model, *n* is the number of fitted points, and $\ln (L)$ is the log-likelihood associated with the model.

**Fig. 7:**
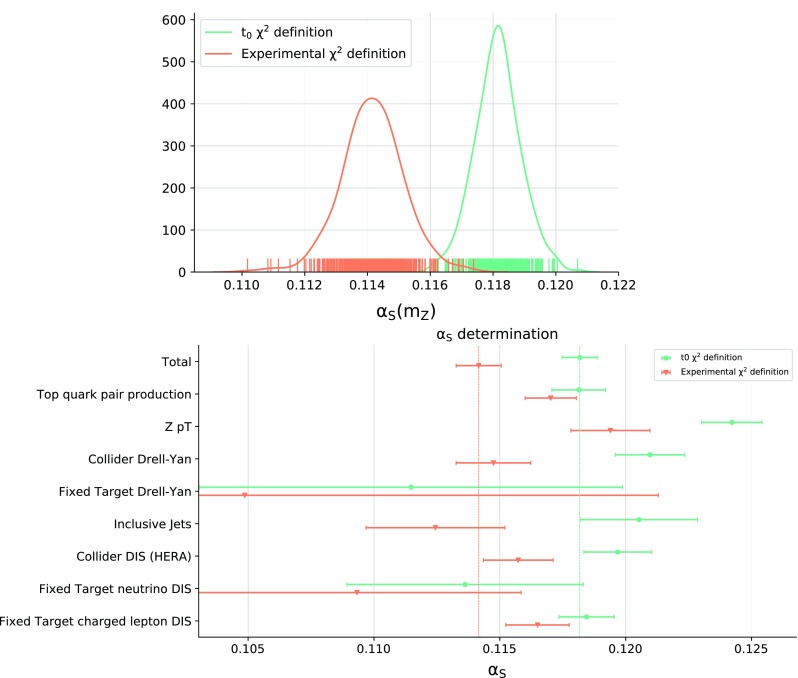
Top: probability distributions for the best-fit $\alpha _s^{(k)}$ values (same as Fig. 3) and bottom: values of the partial $\alpha _s\left( m_Z \right)$ and corresponding uncertainties (same as Fig. 5) in both cases comparing NNLO results from a single batch found using either a consistent or an inconsistent definition of the $\chi ^2$

In our case, we fit to $\chi ^{2(k)}(\alpha _s)$, Eq. (2.5), viewed as a function of $\alpha _s$ using either a parabola (as in our default determination) or a higher order polynomial. The log-likelihood is then in each case just the $\chi ^2$ of this fit. Computing the AIC score for each fitted profile, averaging over c-replicas, and taking the variance of results as a measure of the uncertainty, we find $\mathrm {AIC}=169 \pm 37$ for the default quadratic fit and $\mathrm {AIC}=173 \pm 35$ for a cubic fit. We conclude that there is no evidence that a cubic fit is better than a quadratic one.

**Table 5:** Same as Table 2, comparing the default parabolic fitting (in boldface) of the $\chi ^2(\alpha _s)$ profiles with those with a transformed input, both $\chi ^2\left( \ln (1+\alpha _s)\right)$ and $\chi ^2\left( \exp (\alpha _s)\right)$

	$\alpha _s\left( m_Z \right)$	$N_\mathrm{rep}$
**default**	$\mathbf{0.11845 \pm 0.00052~(0.4\%)}$	**379**
ln	$0.11845 \pm 0.00052~(0.4\%)$	379
exp	$0.11849 \pm 0.00052~(0.4\%)$	379

We perform a final test based on the observation that any transformation of the error function profile of the form

3.5 $$\begin{aligned} \chi ^{2}(\alpha _s)\rightarrow \chi ^{2}(f(\alpha _s)), \end{aligned}$$

where *f* is sufficiently smooth and monotonic, should lead to the same best-fit value of $\alpha _s$. The results of fitting $\alpha _s$ from the transformed profiles Eq. (3.5) with $f(\alpha _s)=\exp (\alpha _s)$ and $f(\alpha _s)=\ln (1+\alpha _s)$ are shown in Table 5. The argument of the log is shifted so that $f(\alpha _s)$ admits a Taylor expansion in powers of $\alpha _s$.

Reassuringly, we find extreme stability with respect to these transformations of the fitting argument.

Combining results from Tables 4 and 5 and the analysis based on the AIC score we can conservatively take as an estimate of the uncertainty related to parabolic fitting the largest shift observed in Table 2, neglecting the cases with $N_\mathrm{rep}<100$ which are dominated by finite-size uncertainty, namely

3.6 $$\begin{aligned} \Delta _\mathrm{par}=0.00010~(0.08\%). \end{aligned}$$

We finally turn to the uncertainty related to the treatment of experimental correlated systematic errors. As mentioned in Sec. 2.1, the covariance matrix in the presence of multiplicative uncertainties should not be identified with the experimental covariance matrix, in order to avoid biasing the fit [23]. We thus adopt the $t_0$ method, introduced in [24], benchmarked in [25], and used for the determination of all NNPDF sets from NNPDF2.0 [40] onwards. In this procedure, the normalization of the multiplicative uncertainties that enter the covariance matrix is iteratively determined from a prior theory prediction. Because the PDFs and $\alpha _s$ are now determined on the same footing, the same covariance matrix is used for both. It is clear that the same $\chi ^2$ definition must be used in Eq. (2.9) as in Eqs. (2.7)–(2.8) in order for the same minimum to be found.

Indeed, it is interesting to note that using an inconsistent definition of the covariance matrix significantly biases the result of $\alpha _s\left( m_Z \right)$. In Fig. 7 we compare the distribution of NNLO $\alpha _s\left( m_Z \right)$ values as well as the total and partial best-fit values and uncertainties, computed for a single batch, either consistently using the $t_0$ covariance matrix (see Figs. 3, 5 for the corresponding results with three batches) or inconsistently using the experimental covariance matrix. We find that the inconsistent definition leads to a much broader distribution for the total $\chi ^2$, thereby signaling the lack of consistency, and, more importantly, a biased central value $\alpha _s(m_Z) = 0.114 \pm 0.001^\text {exp}~(0.9\%)$, shifted by about 9-$\sigma$ in comparison to the correct result Eq. (3.1). The fact that a downward shift of $\alpha _s\left( m_Z \right)$ is observed when using the inconsistent definition can be understood based on the observation that the bias [23] typically leads to the best-fit undershooting the data, essentially because with multiplicative uncertainties a lower prediction has a smaller uncertainty [41]. Indeed, inspection of the partial best-fit values shows that the bias is much stronger for collider experiments than the fixed-target ones. This is what one would expect, because systematic uncertainties are multiplicative for collider experiments, while they are mostly additive for fixed-target [25], so any effect or bias related to the treatment of multiplicative uncertainties should be mostly seen in collider data.

**Table 6:** Best-fit value of $\alpha _s\left( m_Z \right)$ and experimental uncertainty found using three different forms of the $t_0$ covariance matrix (see text); the second row corresponds to the central result Eq. (3.1). The number of c-replicas selected in each case is also shown

$t_0$	$\alpha _s\left( m_Z \right)$	$N_\mathrm{rep}$
I	$0.11844 \pm 0.00052 (0.4\%)$	379
II	$0.11845 \pm 0.00052 (0.4\%)$	379
III	$0.11841 \pm 0.00051 (0.4\%)$	356

The use of the $t_0$ procedure in principle leads to a further methodological uncertainty related to the choice of the prior used for the construction of the $t_0$ matrix, which should therefore be assessed. In order to determine the final result Eq. (3.1) the $t_0$ matrix was constructed using the best-fit PDF set from batch II of Table 3. We have repeated the determination constructing the $t_0$ matrix from the best-fit PDF set of either of the other two batches. Results are collected in Table 6. It is clear that, using the consistent $t_0$ method, results are extremely stable. We can conservatively estimate the uncertainty due to the choice of $t_0$ from the largest shift seen in Table 6 as $\Delta _{t_0}=0.00004~(0.03\%)$.

In summary, we conservatively estimate methodological uncertainties by adding in quadrature the finite-size uncertainty $\Delta _{\alpha _s}=0.00003$, the uncertainty related to the parabolic approximation $\Delta _\mathrm{par}=0.00010$ and the uncertainty related to the treatment of correlated systematics $\Delta _{t_0}=0.00004$, with the result

3.7 $$\begin{aligned} \sigma ^\text {meth} = 0.00011~(0.09\%). \end{aligned}$$

Therefore, we find that, at NNLO, methodological uncertainties are smaller than the experimental uncertainties Eq. (3.1) by a factor five.

### Theoretical uncertainties from missing higher orders

A determination of $\alpha _s\left( m_Z \right)$ is dependent on the perturbative order of the QCD calculations on which it relies. Therefore, at any fixed order it is affected by a missing higher order uncertainty (MHOU). In older, and also some more recent determinations of $\alpha _s\left( m_Z \right)$ (specifically for determination in PDF fits see Refs. [17, 42, 43]) no attempt was made to estimate the MHOU, and sometimes NLO or NNLO values of $\alpha _s\left( m_Z \right)$ were quoted with the understanding that they might differ by an amount greater than the quoted uncertainty due to this missing uncertainty. However, as the experimental uncertainty decreases, an estimate of the MHOU becomes mandatory, and in the context of PDF fits it was done e.g. in Ref.  [18]. Indeed, this uncertainty, usually estimated by scale variation, is typically dominant in more recent determinations [9–15].

In the present case, a first handle on the MHOU associated to $\alpha _s$ is provided by the difference between the NLO and NNLO results Eqs. (3.1) and (3.2), namely

3.8 $$\begin{aligned} \Delta \alpha _s^\text {pert} \equiv | \alpha _s^\text {NNLO} - \alpha _s^\text {NLO} | = 0.0022, \end{aligned}$$

which corresponds to a $2\%$ shift of the NNLO central value. This is about four times larger than the experimental uncertainty in Eq. (3.1), thereby suggesting that even at NNLO the MHOU on the $\alpha _s\left( m_Z \right)$ determination might be comparable to, or larger than the experimental uncertainty.

In our previous determination of $\alpha _s$ Ref. [18] the MHOU was estimated using the Cacciari–Houdeau (CH) method [44], which relies on a Bayesian estimate of the missing higher perturbative orders based on the behaviour of the known orders. Use of exactly the same method of Ref. [18], to which the reader is referred for details, leads to the values

3.9 $$\begin{aligned} \Delta ^\text {CH, NLO}&= 0.003 , \end{aligned}$$

3.10 $$\begin{aligned} \Delta ^\text {CH, NNLO}&=0.0004, \end{aligned}$$

for the 68% confidence level MHOU on $\alpha _s(M_Z)$. The rather large difference in the MHOU estimate between NLO and NNLO stems from the fact that there is a significant shift when going from LO to NLO, but a much smaller one when going from NLO to NNLO.

The NLO estimate of the MHOUs in Eq. (3.9) is reassuringly in good agreement with the observed shift Eq. (3.8). The NNLO uncertainty Eq. (3.10) is also consistent with expectations based on the CH uncertainty estimate of Ref. [18], where the value of $\alpha _s\left( m_Z \right)$ determined using the NNPDF2.1 set was found to lead to $\Delta ^\text {CH, NNLO}=0.0009$. Indeed, PDF uncertainties in the NNPDF3.1 set are generally smaller than those on NNPDF2.1 by a factor of two or more, due to significant impact of LHC data in the more recent determination.

In addition, the shift between NLO and NNLO PDFs is found to be smaller in NNPDF3.1 than in previous NNPDF sets [45], presumably because MHO terms pull in different directions and thus partly cancel each other to a greater extent in a more global fit. Indeed, we find a similar increase of perturbative stability of PDFs and of the associated $\alpha _s\left( m_Z \right)$ by repeating the analysis presented here for reduced datasets [46]. Therefore, the reduction of the MHOU by a comparable factor in Eq. (3.8) in comparison to Ref. [18] is expected.

Nevertheless, the very small value of the MHOU at NNLO, Eq. (3.10), even smaller than the already small experimental uncertainty Eq. (3.1), may seem rather too optimistic. There are furthermore several reasons of principle and practice why the reliability of the CH method in the present case is dubious. The main one is that the implementation of the method suggested in Ref. [18] relies on a guess for an underlying “true” value $\alpha _s^{(0)}$, and for a leading-order value $\alpha _s^\text {LO}$, neither of which is known. The result Eqs. (3.9–3.10) is obtained by varying $\alpha _s^\text {LO}\in [0.10,0.14]$. and $\alpha _s^{(0)}\in [0.110,0.125]$. These are, however, largely arbitrary choices, and the final answer relies on them.

We therefore prefer to adopt a more conservative estimate. Namely, we assume that the MHOU on the NNLO result is half the difference between the NLO and NNLO results Eq. (3.8):

3.11 $$\begin{aligned} \Delta \alpha ^\mathrm{th}_s =0.0011~(0.9\%), \end{aligned}$$

about twice the size of the corresponding experimental uncertainty Eq. (3.1). Whereas this is surely a very crude estimate, we do not feel that any of the available methods can lead to a more reliable conclusion.

On top of the missing higher fixed-order QCD corrections, several other aspects of the theory used in the simultaneous determination of $\alpha _s\left( m_Z \right)$ and PDFs also lead to uncertainties. These include the values of the heavy quark masses, standard model parameters (specifically CKM matrix elements and electroweak couplings), electroweak corrections, QCD resummation corrections [47, 48], QCD power corrections, and nuclear corrections. Many of these uncertainties were assessed in the NNPDF3.1 PDF determination that we are relying upon [16], and found to be smaller than PDF uncertainties. In particular, the dependence on the charm mass in previous PDF determinations is substantially reduced in NNPDF3.1 and likely rather smaller than the MHOU, thanks to the presence of an independently parametrized charm PDF [49], and electroweak corrections are carefully kept under control thanks to the choice of suitable kinematic cuts. But PDF uncertainties mix with the experimental uncertainty on $\alpha _s\left( m_Z \right)$, with which they are strongly correlated, and are in fact indistinguishable from it, as discussed in Sect. 2.1, so the hierarchy of uncertainties on PDFs and $\alpha _s\left( m_Z \right)$ is the same. We conclude that we have evidence that most of these theoretical uncertainties are sub-dominant in comparison to the experimental uncertainty Eq. (3.1), and thus even more so in comparison to the MHOU Eq. (3.11).

### Final results and comparisons

We can now collect results. Combining the NNLO value and experimental uncertainty Eq. (3.1), the methodological uncertainty Eq. (3.7) and the theoretical uncertainty Eq. (3.11) we get

3.12 $$\begin{aligned} \alpha _s^\text {NNLO}(m_Z)= & {} 0.1185 \pm 0.0005^\mathrm{exp}\pm 0.0001^\mathrm{meth}\pm 0.0011^\mathrm{th}\nonumber \\= & {} 0.1185 \pm 0.0012~(1\%), \end{aligned}$$

where in the last step we have added all uncertainties in quadrature. For a comparison to other determinations, such as the PDG average, we recommend using only the experimental uncertainty (the methodological uncertainty being negligible), which reflects the limitations of our result and procedure, but not the limitation due to the fact that our result is obtained at NNLO. For precision phenomenology, however, we recommend use of the total uncertainty in order to conservatively account for the MHOU.

This result can be compared to the previous one [18] based on NNPDF2.1, $\alpha _s^\text {NNLO}(m_Z) = 0.1173 \pm 0.0007^\text {exp}\pm 0.0009^\text {th}$. In comparison to this older result, the central value of $\alpha _s(m_Z)$ has increased by $\Delta \alpha _s=+0.0012$ . As far as uncertainties are concerned, both the theoretical and experimental uncertainties on this previous result are larger, if one compares like with like. The experimental uncertainty should actually be compared to Eq. (3.3) as it was obtained with the same method. The uncertainty is somewhat underestimated because it neglects the correlation between PDFs and $\alpha _s$, while the theory uncertainty should be compared to Eq. (3.10) which is also based on the CH method.

We conclude that, in comparison to Ref. [18], the current result is more precise, though with more conservatively estimated uncertainties.

**Fig. 8:**
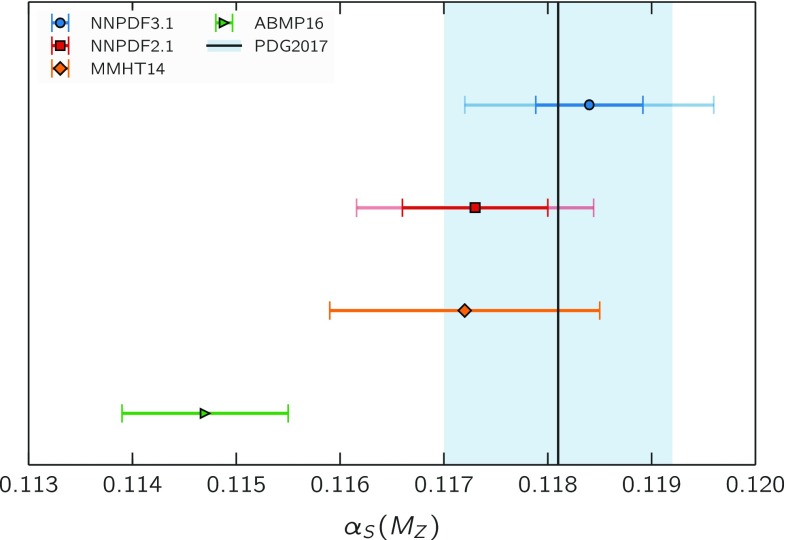
Comparison of the present NNLO determination of $\alpha _s\left( m_Z \right)$, Eq. (3.12), with the PDG average and with the previous ABMP16, MMHT14, and NNPDF2.1 results. For the NNPDF values, the inner (darker) error bar correspond to experimental uncertainties, while the outer (lighter) one indicates the sum in quadrature of experimental and theoretical uncertainties

In Fig. 8 we compare the NNLO result of Eq. (3.12) to our previous result [18], to the current PDG average [3], and to two recent determinations obtained from simultaneous fit of PDFs and $\alpha _s\left( m_Z \right)$, ABMP16 [43] and MMHT2014 [42]. We find good agreement with the PDG average as well as with the MMHT14 and NNPDF2.1 determinations. It has been suggested [50, 51] that the lower ABMP16 value can be partly explained by the use of a fixed-flavour number scheme with $N_f=3$ for the treatment of DIS data. It is interesting to observe that the current AMBP16 value is higher than previous values of $\alpha _s\left( m_Z \right)$ obtained by the same group [52], from which the ABMP16 analysis in particular differs because of inclusion in Ref. [43] of LHC top production and *W* and *Z* production data (described with $N_f=5$).

Interestingly, the $\alpha _s\left( m_Z \right)$ determination from the NNPDF3.1 fit is higher than any other recent determination from PDF fits. Inspection of Figs. 4 and 6 strongly suggests that this increase is driven by the high-precision LHC data, especially for gauge boson production (including the *Z* $p_T$ distribution) but also for top and jet production.

## Summary and outlook

In this work we have presented a new determination of the strong coupling constant $\alpha _s\left( m_Z \right)$ jointly with a global determination of PDFs which, by relying on NNPDF3.1, for the first time includes a large amount of LHC data using exact NNLO theory in all cases. In comparison to a previous determination based on NNPDF2.1, our results exploit the new correlated replica method that is equivalent to the simultaneous fit of PDFs and $\alpha _s$. This new method thus fully accounts for the correlations between PDFs and $\alpha _s$ in the determination of the best-fit value of $\alpha _s$ and of the associated uncertainty.

We find that the determination of $\alpha _s\left( m_Z \right)$ is considerably stabilized by the use of a wide set of different processes and data, and we provide evidence that a global simultaneous determination of $\alpha _s\left( m_Z \right)$ and PDFs leads to a more stable and accurate result than the one obtained from subsets of data. We thus obtain a value of $\alpha _s\left( m_Z \right)$ which is likely to be more precise and more accurate than previous results based on similar techniques. We find that the LHC data consistently lead to an increase in the central value of $\alpha _s\left( m_Z \right)$, and observe good overall consistency between the datasets entering the global fit. Our NNLO determination turns out to be in agreement within uncertainties with previous results from global fits and with the PDG average.

The main limitation of our result comes from the lack of a reliable method to estimate the uncertainties related to missing higher order perturbative corrections. Theoretical progress in this direction is needed, and perhaps expected, and would be a major source of future improvement. For the time being, even with a very conservative estimate of the theoretical uncertainty, our result provides one of the most accurate determinations of $\alpha _s\left( m_Z \right)$ available, and thus provides valuable input for precision tests of the Standard Model and for searches for new physics beyond it.
